# Evaluation of intermediate care for knee and hip osteoarthritis: a mixed-methods study

**DOI:** 10.1186/s12875-021-01474-0

**Published:** 2021-06-24

**Authors:** Ilgin G. Arslan, Vincent M. I. Voorbrood, Saskia A. G. Stitzinger, Maarten-Paul van de Kerkhove, Rianne M. Rozendaal, Marienke van Middelkoop, Patrick J. E. Bindels, Sita M. A. Bierma-Zeinstra, Dieuwke Schiphof

**Affiliations:** 1 Department of General Practice, Erasmus MC University Medical Center, P.O. Box 2040, Rotterdam, 3000 CA The Netherlands; 2 General Practice Pallion, Hulst, The Netherlands; 3 Orthopaedics ZorgSaam Zeeuws-Vlaanderen, Terneuzen, The Netherlands; 4 Department of Orthopaedics, Erasmus MC, University Medical Center, Rotterdam, The Netherlands

**Keywords:** Intermediate care, Joint consultations, General practice, Knee osteoarthritis, Hip osteoarthritis

## Abstract

**Background:**

To evaluate intermediate care for knee and hip osteoarthritis (KHOA) in the general practice that incorporate specialist services into general practice to prevent unnecessary referrals to hospitals.

**Methods:**

We used a mixed methods approach including semi-structured interviews, patient experience questionnaires and data from medical records from three intermediate care projects. Semi-structured interviews were conducted with patients, general practitioners (GPs), orthopaedists and a healthcare manager in intermediate care. Satisfaction of patients who received intermediate care (*n* = 100) was collected using questionnaires. Referral data and healthcare consumption from medical records were collected retrospectively from KHOA patients before (*n* = 96) and after (*n* = 208) the implementation of intermediate care.

**Results:**

GPs and orthopaedists in intermediate care experienced more intensive collaboration compared to regular care. This led to a perceived increase in GPs’ knowledge enabling better selection of referrals to orthopaedics and less healthcare consumption. Orthopaedists felt a higher workload and limited access to diagnostic facilities. Patients were satisfied and experienced better access to specialists’ knowledge in a trusted environment compared to regular care. Referrals to physiotherapy increased significantly after the implementation of intermediate care (absolute difference = 15%; 95% CI = 7.19 to 22.8), but not significantly to orthopaedics (absolute difference = 5.9%; 95% CI = -6.18 to 17.9).

**Conclusions:**

Orthopaedists and GPs perceived the benefits of an intensified collaboration in intermediate care. Intermediate care may contribute to high quality of care through more physiotherapy referrals. Further research with longer follow-up is needed to confirm these findings and give more insight in referrals and healthcare consumption.

**Supplementary Information:**

The online version contains supplementary material available at 10.1186/s12875-021-01474-0.

## Background

Osteoarthritis is one of the most prevalent chronic diseases, affecting 250 million people worldwide; the knees and hips are the most affected joints [1]. The prevalence and disability burden of osteoarthritis are increasing [2, 3]. As a consequence, healthcare costs are increasing dramatically. Hospital care accounts for the biggest component of healthcare costs, with knee and hip replacements being a substantial element [1, 3–5]. Previous studies have shown that inappropriate joint replacements are common before core treatments for osteoarthritis (e.g. self-management education and exercise therapy [6]) have been optimally used. This leads to high unnecessary healthcare costs [7, 8]. Initiatives to address this rise in healthcare costs and hospital overuse are needed to support the affordability of the healthcare system [9, 10].

To improve access to specialist services, reduce demand on hospitals, and enhance relationships between primary care providers and medical specialists, several ‘shifted outpatients’ models have been developed [11, 12]. These models focus on the substitution of hospital-based specialist care into a primary care setting, for example by replacing the primary care provider with a medical specialist as the doctor of first contact (i.e. ‘replacement model’), or strengthening the relationship between medical specialists and primary care, but with most patient care mediated through the general practitioner (GP) (i.e. “consultation” model), or with the medical specialist as part of a team of visiting services (i.e. “liaison attachment” model) [11–13]. In the Netherlands, GPs act as a gatekeeper to secondary care (i.e. hospital services) and patients can only access hospital services by a referral from their GP [14, 15]. Reinforcement of this gatekeeping role of GPs may help prevent unnecessary referrals to hospitals and thereby tackle rising healthcare costs.

A Dutch nation-wide initiative started in 2012 after the Dutch Ministry of Health, Welfare and Sport formulated recommendations to slow down rising costs through substitution of hospital care to primary care with care provided at ‘the right place’. Based on this recommendation, an agreement was made between the Ministry of Health, Welfare and Sport and the National General Practitioners Association to investigate whether substitution of hospital care to primary care can be introduced in the Dutch healthcare system [16]. Therefore, a relatively new outpatient model was initiated in the Netherlands, termed ‘intermediate care’, often in the form of one-time consultations by medical specialists in the general practice [17, 18]. Previous research has shown the value of intermediate care for reducing waiting times in several medical specialties (e.g. dermatology, orthopaedics, cardiology and rheumatology) [17]. However, studies that evaluated the effect on referrals to hospitals are scarce and to date have not included knee and hip osteoarthritis (KHOA) [17]. Evidence regarding intermediate care for KHOA is urgently needed as KHOA accounts for a large proportion of hospital overuse and rising healthcare costs.

Recently, in cooperation with health insurance companies, several pragmatic pilot projects have started in the Netherlands to implement intermediate care for KHOA in general practices. Within these projects, orthopaedists (i.e. orthopaedic surgeons) provided face-to-face consultations in general practices. We evaluated three of these projects with regard to: 1) facilitators and barriers of intermediate care as perceived by patients and stakeholders; 2) patient satisfaction; and 3) the effect on the number of referrals to orthopaedics and physiotherapy, and healthcare consumption.

## Methods

Intermediate care projects in three general practices initiated by the Dutch health insurance company CZ agreed to participate (Practices A-C). Practices A and C are located in an urban area with intermediate care constructed as a one-time consultation by an orthopaedist (i.e. orthopaedic surgeon) in the general practice to patients with musculoskeletal complaints. Practice B is located in a rural area and provided joint consultations by an orthopaedist and GP to patients with KHOA. At the start of this evaluation study, the projects in practices A and B had been running for one year, and the project in practice C for two years.

A mixed methods approach was performed using semi-structured interviews and data from medical records from the general practices. Practices A and B had already collected data on patient satisfaction, which we also included in the current study. Although practices A and C provided intermediate care to patients with all types of musculoskeletal complaints, this study focused on intermediate care provided to the subgroup of patients with KHOA. The characteristics and the parts of the evaluation programme that the projects participated in are shown in Table 1.

**Table 1:** Participating practices and their characteristics

	**Practice A**	**Practice B**	**Practice C**
**Target group**	Patients with musculoskeletal complaints who would normally be referred to secondary care	1) Patients with suspected knee or hip osteoarthritis 2) Patients with knee or hip osteoarthritis aged 50 and older that do not qualify for surgery and patients who do not sufficiently respond to non-surgical treatment in primary care	Patients with musculoskeletal complaints who would normally be referred to secondary care
**Area**	Urban	Rural	Urban
**Healthcare providers in intermediate care consultation**	Orthopaedist, sometimes together with a GP	Joint consultation by a GP and orthopaedist	Orthopaedist
**Total number of healthcare providers involved in the project**	One GP and one orthopaedist	Three GPs, two orthopaedists, and one healthcare manager	Two GPs and one orthopaedist
**Scale of project**	One general practice with one orthopaedist	Two general practices; one orthopaedist within each practice	One general practice with one orthopaedist
**Participated in following programme evaluation parts**	Semi-structured interviews, patient-level referral data and patient-reported experience measures	Semi-structured interviews, patient-level referral data and experience measures	Patient-level referral data
**Data that had already been collected by the practice before the start of this evaluation study**	Patient-reported experience measures (patient satisfaction)	Patient-reported experience measures (patient satisfaction)	-

### Semi-structured interviews

All healthcare providers (GPs (*n* = 4), orthopaedists (*n* = 3) and healthcare managers (*n* = 1)) providing intermediate care in practices A and B were invited for semi-structured interviews. Non-responders received a reminder within 2 weeks of the invitation. These interviews focused on their perceived facilitators and barriers with intermediate care. In addition, GPs were asked to invite a convenience sample of patients with KHOA who had at least one intermediate care consultation to be interviewed. These interviews included pre-determined topics from the literature and based on the expert opinion of the research group. Based on these topics, interview guides with open-ended questions were composed and pilot tested (Supplementary Table 1). One researcher (IGA, physiotherapist and researcher) conducted the interviews face-to-face in the general practice or by telephone. The pre-determined topics needed to be covered during the conversations, although the interviewer was allowed to diverge from the interview guide to explore additional topics. Field notes were made by the interviewer during and after the interviews. The interviews were audiotaped, transcribed verbatim into written form and read by the interviewer to increase the validity. To guarantee transparency, all participants were offered to receive their transcript for comment and correction.

### Patient satisfaction

We included satisfaction questionnaires that were already designed and collected by GPs and orthopaedists from practices A and B. Immediately after the intermediate care consultation, patients were asked by the GP assistants to complete the questionnaire anonymously. These questionnaires included questions about satisfaction with: 1) the consultation; 2) the provision of information by healthcare providers; and 3) the patient-healthcare provider relationship. Satisfaction scales in the questionnaires varied between the two practices (1–10 scale vs. 4-point Likert scale). Patients were allowed to add free-text comments.

### Healthcare consumption and referrals

**Fig. 1:**
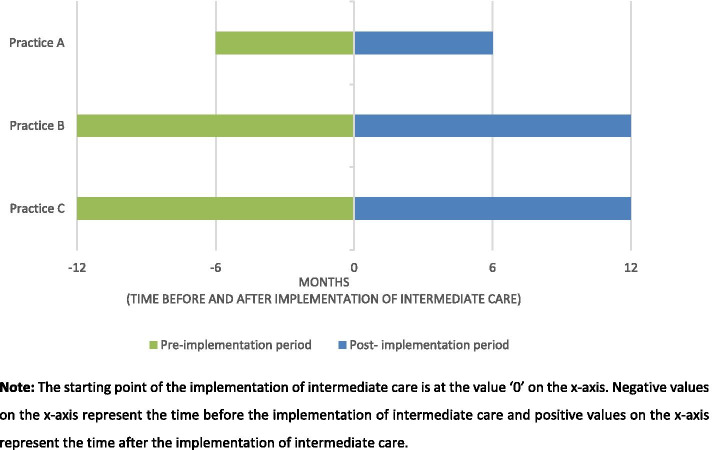
Time periods of the pre-implementation and post- implementation period of intermediate care for data collection on referrals

Data on patients’ characteristics (e.g. age and sex), healthcare consumption in terms of number of consultations (i.e. face-to-face consultation, visit and telephone contact) and GP referrals to orthopaedics in hospital care were collected retrospectively from the medical records of practices A-C. In the Dutch healthcare system, GPs can refer patients to primary care physiotherapists, which is recommended by the Dutch GP guidelines for non-traumatic knee complaints [19] as part of OA core treatment [15, 19]. GP referrals to primary care physiotherapy were also collected retrospectively from the medical records of practices A-C. Records of patients with KHOA and with at least one consultation, visit or telephone contact by their GP either before or after the implementation of intermediate care (pre-implementation and post-implementation period) were selected. A diagnosis of KHOA was defined following the International Classification of Primary Care [20], coding L89 (hip osteoarthritis) and/or L90 (knee osteoarthritis). The duration of the pre- and post-implementation periods varied between the practices, since the practice projects were running for different periods at the time of this study. In practice A data was collected six months before and after implementation (i.e. one year in total) and in practices B and C one year before and after implementation (i.e. two years in total). Figure 1 shows the time periods for the practices in the pre-and post-implementation periods.

### Data analysis

Interviews were analysed following the steps of thematic analysis described by Braun and Clarke and with assistance of the software MAXqda Version 2018 [21, 22]. We used a semantic approach (i.e. analysis close to participants’ language, capturing explicit meaning) to describe the opinions of different participants in intermediate care. Deductive coding was done based on pre-determined themes, and inductive coding based on additional topics that we extracted from the open-ended questions. Two interviews were coded independently by two researchers (IGA and DS) and then compared. Any disagreements were discussed until consensus was reached. The resulting codes were further applied for analysis, and iteratively modified if necessary after each interview coding. Codes were structured hierarchically and analysed using the thematic framework. Relevant quotes were selected from the transcripts [22, 23].

Descriptive statistics of patient satisfaction, healthcare consumption (i.e. number of consultations) and referral data were analysed using R Studio Software V.3.6.3. Means and standard deviations (SDs) were calculated for numeric variables, and numbers (n) and percentages (%) for categorical variables. Differences in the percentage of referrals were assessed using the Chi-squared test with Yates’s continuity correction. Absolute differences in the percentage of referred patients were reported, including 95% confidence interval (CI). The significance level throughout was set at two-tailed *P* < 0.05.

## Results

### Semi-structured interviews

Four GPs, two orthopaedists, and one healthcare manager were interviewed. Only one orthopaedists did not respond to the invitation letter and was therefore not interviewed. Furthermore, four patients invited by their GP were interviewed (see Supplementary Table 2 for their characteristics). The duration of the interviews varied from 7.5 to 45 min. Information about the content of the projects that we extracted from the interviews is presented in Supplementary Table 3.

#### Facilitators for general practitioners, orthopaedists and healthcare manager

GPs and orthopaedists experienced better multidisciplinary communication, with more transparency and mutual respect. They perceived this as an advantage for patients’ trust in the healthcare system and for the relationship between healthcare providers (Quote 2, Table 2). In addition, GPs experienced an increase in their skills and more confidence about their clinical diagnosis. They also perceived more knowledge regarding indications of patients’ referral to secondary care due to intensive multidisciplinary collaboration. This was reported more frequently in projects with joint consultations by an orthopaedist and GP (Quotes 2 and 3, Table 2).

**Table 2:** Facilitators and barriers of intermediate care with example quotes

Main themes	Subthemes	Example quotes
**Facilitators**		
Facilitators for healthcare providers	Relationship between healthcare providers: 1) better multidisciplinary communication 2) more mutual respect	***Quote 1:**** “And the specialist is more aware of the problems the GP actually has. In other words, you end up respecting one another more. That’s also an objective I actually find quite important: that you have respect for one another and the patient can see that. If the specialist says, ‘Go back to your GP; what he says is right’, or if I say, ‘This specialist is really good with this particular problem’. And you say that about one another, which gives the patient more confidence too.” (interview 10, GP)*
	Learning effect of healthcare providers: 1) more competent in specific skills 2) more confident about their clinical diagnosis 3) more knowledge about patients’ referral	***Quote 2:**** “You educate one another a bit. I learn from the GP and [the GP] learns from us.”(Interview 5, orthopaedist)* ***Quote 3:**** “But when you have someone sitting next to you who does an awful lot, you start doing it more often too. You see that happening with the knees. Giving an injection in the knee isn’t so difficult, but if you aren’t doing that and you don’t have someone sitting next to you who does it at some point, then you don’t start doing it yourself.” (interview 6, GP)*
Facilitators for patients	Better access to healthcare	***Quote 4:**** “Right, I reckon that patients – certainly older patients – can get there on their own. They don’t need to find someone who can take them to the hospital. Certainly for older patients: they don’t need to find someone who can take them to the hospital.” (Interview 2, orthopaedist)*
	Healthcare in familiar environment on a small scale	***Quote 5:**** “It’s a more pleasant environment because it’s familiar.” (Interview 9, patient)*
	More specialized care	***Quote 6:**** “That sense of involvement with the orthopaedist. Of course you’re more in his field of expertise. The GP is a bit more of a generalist, after all.” (Interview 4, patient)*
	Longer consultations	***Quote 7:**** “That’s precisely what I like about it: the fact that you have more time. And that’s exactly what all the patients say. The fact that there’s plenty of time for the explanation is something that everyone really likes. […] Right, well, you have… I think your contact with the patient is rather more intensive. Of course, that’s because you have more time.” (Interview 2, orthopaedist)*
Facilitators for society as a whole	Lower healthcare costs due to less healthcare consumption	***Quote 8****: “I also think […] that the extra time […] that I have for a patient in the GP practice means that I don’t see the same people coming back so soon. Because I can really explain things properly to them in one go.” (Interview 2, orthopaedist)* ***Quote 9:**** “We educate one another in that regard too, so if there are pointless examinations, we say ‘Don’t do that’. […] It’s also very much a learning process, and we’re going to end up with fewer diagnostic tests.” (Interview 5, orthopaedist)*
	Lower healthcare costs due to better selection of patients for secondary care	***Quote 10:**** “Yes, we’ve been able to keep more than 80 per cent [of the patients] in primary care. Assuming you start with 100 per cent, then an expensive hospital treatment product would have been initialized for all of them and we’ve now managed to prevent that for four fifths.” (Interview 11, healthcare manager)*
**Barriers**		
Less access to additional diagnostic facilities for orthopaedists	-	***Quote 11:**** “A minus point for orthopaedics in an intermediate care project is that you often don’t have access to additional examinations. So you don’t have any X-rays and if someone comes in and you’re thinking it could be osteoarthritis, you’ll still need… to see that, you’ll still need to have an X-ray* *Interviewer: “And what impact does that have for you in your work – the fact that you can’t easily get the additional diagnostics?”* *“Well, it means you still, um, you still end up with people coming back one more time. And so you hesitate just that little bit, as it were, before making the definite diagnosis.” (Interview 2, orthopaedist)*
Workload for orthopaedists	Workload in secondary care	***Quote 12:*** “*That* [not being the case] *has to do with the big wave* [of osteoarthritis patients] *we are now facing. You can simply see it coming now. So we’re getting just as many people now, but we’re seeing more severe cases. The more minor cases are fortunately staying with the GPs for longer.”(Interview 5, orthopaedist)* ***Quote 13:**** “They are seeing an increase in complex care needs. The contamination* [hospital overuse] *that you basically get rid of, because that’s the intermediate care, you are taking that away. And the better care ends up in the right place, so it’s really a reciprocal process.” (Interview 10, healthcare manager)* ***Quote 14:**** “I think that as doctors and specialists, we need to look at whether we shouldn’t perhaps be allocating more time for that patient visiting the outpatient clinic. Because if that’s a more severe case, they’ll need more explanation.”(Interview 3, orthopaedist)*
	Additional workload in general due to intermediate care	***Quote 15:**** “It* [working in intermediate care in addition to working in a hospital] *is busy so that means you have to organize it well. I always do that on my free afternoon. […] There is more pressure on you, quite apart from organizing the whole intermediate care consultations and it takes an awful lot of time. (Interview 5, orthopaedist)*

#### Facilitators for patients

Patients and healthcare providers said that the shorter waiting times, lower out-of-pocket costs and shorter travel distances resulted in better access to healthcare, especially for elderly patients (Quote 4, Table 2). Patients experienced added value in the fact that they received specialist care in a trusted environment on a small scale (i.e. the general practice) (Quotes 5 and 6, Table 2). Healthcare providers benefited from the longer consultations in intermediate care compared to regular care by having more time to inform patients properly about their health problem. They felt that this was highly valued by patients. (Quote 7, Table 2).

#### Facilitators for society as a whole

As a result of the longer consultations, healthcare providers experienced less follow-up consultations in which patients ask for more information compared to regular care (Quote 8, Table 2). Orthopaedists and the healthcare manager experienced less unnecessary diagnostic procedures (e.g. less MRI requests in general practice) due to increasing knowledge of healthcare providers through intensified multidisciplinary communication (Quote 9, Table 2). Furthermore, fewer patients were unnecessarily referred to the hospital (Quote 10, Table 2). Healthcare providers mentioned that this reduction in healthcare consumption led to lower healthcare costs, which benefits society as a whole.

#### Barriers for healthcare providers

Orthopaedists working in intermediate care had limited access to additional diagnostic equipment (e.g. MRI or X-ray equipment). As a consequence, they felt that requesting additional diagnostic tests led to logistics barriers and uncertainties about their diagnosis (Quote 11, Table 2). As a solution, GPs in one project started requesting X-rays routinely before referring patients to intermediate care.

Although orthopaedists agreed that better selection of patients to hospitals is a valuable consequence of intermediate care, some feared that the reduction of referrals to hospitals threatened the hospital’s income. However, this did not appear to be the case, probably because of the increasing prevalence of patients with KHOA (Quote 12, Table 2). Orthopaedists believed that intermediate care reduced the number of referrals to hospitals, as a result, they felt that patients referred to hospitals were more complex and time-consuming patients than before. As a consequence, they felt an increase in their workload in the hospital (Quote 13, Table 2). As complex patients need more information and their healthcare takes more organizing, healthcare providers recommended having longer consultations and employing more support personnel in hospitals (Quote 14, Table 2). Orthopaedists also experienced a higher workload, as the intermediate care project was an additional service on top of their usual work in the hospital (Quote 15, Table 2).

### Patient satisfaction

In total, 100 patients from practices A and B completed the satisfaction questionnaires (data shown in Supplementary Figure 1). Results from practice A (*n* = 39) showed that most patients were ‘very satisfied’ with the consultation (63%), provision of information by healthcare providers (67%), and the patient-healthcare provider relationship (72%) (4-point Likert scale ‘very unsatisfied’ to ‘very satisfied’). The remaining patients were ‘satisfied’. Results from practice B (*n* = 61) showed a mean satisfaction score of 9 (range 0 to 10) for the patient-healthcare provider relationship and provision of information, and 8.9 for the consultation in general. Specific comments about the intermediate care consultation were positive, for example about the knowledge of the orthopaedist, short waiting times and consultation hours in the evening.

### Healthcare consumption and referrals

A total of 96 patients with KHOA were seen during the pre-implementation period and 208 during the post-implementation period. Of the patients in the post-implementation period, 26.4% received intermediate care and the remaining 73.6% received regular GP care. Patients in the pre-implementation period had a mean age of 71.3 years (SD = 10.8), 66.7% of them were female, 67.7% had knee OA, and the remaining 32.3% hip OA. Patients in the post-implementation period had a mean age of 69.3 years (SD = 9.8), 66.3% of them were female, 65.9% had knee OA, and the remaining 43.1% hip OA. Patients in the pre-implementation period received on average 2.40 consultations (SD = 1.59) and patients in the post-implementation period on average 2.52 consultations (SD = 1.78). These characteristics did not significantly differ between patients in the pre- and post-implementation period (Table 3).

**Table 3:** Characteristics of patients in three general practices with intermediate care projects, comparing pre-implementation and post-implementation groups

	Pre-implementation period (*n* = 96)	Post- implementation period (*n* = 208)	Difference *P*-value
Age, mean (SD)	71.3 (10.8)	69.3 (9.8)	*P* = .11
Female, n (%)	64 (66.7)	138 (66.3)	*P* = 1.00
Knee osteoarthritis coding, n (%)	65 (67.7)	137 (65.9)	*P* = .80
Hip osteoarthritis coding, n (%)	31 (32.3)	71 (34.1)	*P* = .80
Number of consultations, mean (SD)	2.40 (1.59)	2.52 (1.78)	*P* = .53

The percentage of referrals to physiotherapy increased significantly in the post-implementation period compared to pre-implementation (absolute difference = 15%; 95% CI = 7.19 to 22.8). In contrast, the percentage of referrals to orthopaedics increased slightly, but not statistically significant (absolute difference = 5.9%; 95% CI = -6.18 to 17.9) (Table 4). Supplementary Table 4 shows the referrals and number of consultations stratified by patients who received regular GP care and patients who received intermediate care during the post-implementation period.

**Table 4:** Referrals to physiotherapy and orthopaedics in the pre-implementation period compared to post-implementation

	Pre-implementation period (*n* = 96)	Post-implementation period (*n* = 208)	Absolute difference (%) (95% CI)
Referrals to physiotherapy, n (%)	5 (5.21)	42 (20.2)	** + 15.0% (7.19–22.8)**
Referrals to orthopaedics, n (%)	29 (30.2)	75 (36.1)	+ 5.9%; (-6.18–17.9)

Bold: statistically significant at 5% level

## Discussion

### Summary

This evaluation study showed that GPs and orthopaedists experienced more intensive collaboration due to the implementation of intermediate care in general practice. This led to a perceived increase in their knowledge, for the GP enabling a better selection of referrals to orthopaedics and physiotherapy. Patients were satisfied and experienced better access to healthcare, and the benefits of a trusted environment and specialists’ knowledge. The percentage of referrals to physiotherapy increased significantly after the implementation of intermediate care. The observed increase in referrals to physiotherapy contributes to the quality of care, since offering patients with KHOA physiotherapy is an indicator for high quality of care [24]. Healthcare providers experienced better selection of referrals to orthopaedics and less healthcare consumption. However, the actual observed percentage of orthopaedic referrals and the mean number of consultations in the general practice did not decrease after the implementation.

### Strengths and limitations

A strength of the study is the mixed methodology that enabled a comprehensive evaluation of intermediate care with regard to the experiences of patients and other stakeholders, patient satisfaction, and referral trends. However, the findings of this study are subject to several limitations. First of all, the retrospective design of this study led to a lack of proper baseline measurements. This limited our information on for example the severity of KHOA and conclusions about the appropriateness of referrals to orthopaedics and physiotherapy are therefore not possible. It should be noted that the differences in referrals might partly be due to confounding by indication for a referral (e.g. more severe patients may be more likely to be referred to orthopaedics) and not only the effect of intermediate care. We were not able to draw conclusions about the effect of differences in patients’ characteristics between the pre- and post-implementation period on referrals. A regression model which would be appropriate for this kind of analysis requires independent samples, which might not be the case in our study. Nevertheless, explorative analysis showed no effect of age, sex or affected joint on referrals (data not shown). Furthermore, the current study only captured GP referrals to physiotherapy. Since 2006 patients in the Netherlands can also access physiotherapy care without a GP referral [25]. The number of physiotherapy uptake might therefore be underestimated in this study. Also, GPs invited a convenience sample of patients for the interviews and the experiences of those patients were generally positive. However, this may be the result of selection as GPs may have been more inclined to invite patients who are more positive about the provided care. Furthermore, all patients preferred a telephone interview instead of face-to-face interview. This, in addition to the low number of patients included, might have influenced the limited data saturation. As a consequence, findings from the interviews with patients might not be reflective of the full range of patient experience. Lastly, the findings of this study are restricted to intermediate care, a ‘shifted-outpatient’ model specifically in the Netherlands. Therefore, applicability to other countries may be limited.

### Comparison with existing literature

Previous studies have shown that GPs have little confidence in their ability to diagnose and manage musculoskeletal conditions [26, 27]. The present study showed that GPs and orthopaedists providing intermediate care felt that they learned from each other and that their knowledge increased. Therefore, intermediate care might be a solution to increase the confidence of GPs.

Furthermore, this study showed that orthopaedists experienced a higher workload due to intermediate care. Previous research has shown that a substantial proportion of patients referred to secondary care could instead be seen by a GP with special interest in this area [28]. This may therefore be helpful in managing the high workload for orthopaedists and is worth exploring in future research. Orthopaedists also felt they had limited access to diagnostic facilities in the general practices, which is in line with a previous study [29] that evaluated barriers and facilitators in substituting hospital care with primary care. This barrier may lead to an increase in healthcare costs, as GPs in one project started requesting X-rays routinely before referring patients to intermediate care, while current clinical practice guidelines [19] do not recommend routine X-rays in primary care settings. Previous studies reported a decrease in referrals to orthopaedics [11, 13, 29, 30]. However, the current study shows that while healthcare providers experienced a better selection of referrals to orthopaedics, the actual observed percentage of referrals did not decrease. This might be due to the short follow-up time of the intermediate care projects. A longer follow-up time is probably needed to observe more reliable effects of intermediate care on referrals and healthcare consumption.

Healthcare providers who were interviewed in the present study felt that the longer consultation in intermediate care is a benefit for patients. However, a recent study [31] showed that patients did not find the duration of the consultation very important, while healthcare providers did. Our study shows that healthcare providers experience longer consultations as a facilitator for providing better medical advice to patients, which might reduce the patient’s need for further consultations. This finding is in line with results from a previous observational study [32]. Even though the observed mean number of consultations in the current study did not yet decrease, future research with a longer follow-up time may show a reduction.

### Implications for research

We strongly recommend replication of this study with more rigorous data collection methods and a prospective study design (e.g. cluster or stepped wedged randomized controlled trial that decrease potential bias) to increase the reliability of the findings. In addition, a longer follow-up time in future research would be justified to show the long-term effects of intermediate care on referrals and healthcare consumption. Further research including different forms of intermediate care is also needed to provide more extensive recommendations on how to implement intermediate care most effectively, such as electronic consultations between GPs and specialists [33]. Lastly, our research indicated that intermediate care reduces healthcare costs based on the experiences of healthcare providers, as expressed in the interviews. Future research into the cost-effectiveness of intermediate care is recommended to strengthen the evidence for this result.

## Conclusions

This evaluation study of intermediate care for KHOA showed benefits in intensifying the collaboration between orthopaedists and GPs. This led to a perceived increase in their knowledge enabling better selection of referrals to orthopaedics and decrease in healthcare consumption. In contrast, orthopaedists providing intermediate care felt a higher workload and limited access to diagnostic facilities. Patients were satisfied and experienced better access to healthcare and the specialists’ knowledge in a trusted environment. Intermediate care led to an increase in physiotherapy referrals, contributing to high quality of care, but did not reduce the number of referrals to orthopaedics and healthcare consumption in these projects yet.

## Supplementary Information

**Additional file 1: Supplementary Table 1**. Interview guides. **Supplementary Table 2**. Characteristics of participants participating the semi-structured interviews. **Supplementary Table 3**. Content of the intermediate care projects for which semi-structured interviews were carried out. **Supplementary Figure 1**. Patients’ satisfaction with intermediate care. **Supplementary Table 4**. Referrals and healthcare consumption in the pre-and post-implementation period stratified by patients who received regular GP care or intermediate care.

## Data Availability

All data generated or analysed during this study are included in this published article and supplementary files.

## Abbreviations

GP: General practitioner
KHOA: Knee and hip osteoarthritis
SD: Standard deviation
n: Number
%: Percentage
CI: Confidence interval
