# Supplementary material for: Evaluation of intermediate care for knee and hip osteoarthritis: a mixed-methods study
Source: BMC Fam Pract. 2021 Jun 24;22:131. doi: 10.1186/s12875-021-01474-0 (PMC8229342; doi:10.1186/s12875-021-01474-0)
Supplement: Supplementary file 1 — Additional file 1: Supplementary Table 1. Interview guides. Supplementary Table 2. Characteristics of participants participating the semi-structured interviews. Supplementary Table 3. Content of the intermediate care projects for which semi-structured interviews were carried out. Supplementary Figure 1. Patients’ satisfaction with intermediate care. Supplementary Table 4. Referrals and healthcare consumption in the pre-and post-implementation period stratified by patients who received regular GP care or intermediate care. [file 12875_2021_1474_MOESM1_ESM.docx]

**EVALUATION OF INTERMEDIATE CARE FOR KNEE AND HIP OSTEOARTHRITIS: A MIXED-METHODS STUDY**

MSc Ilgin G. Arslan^1^, MD Vincent M.I. Voorbrood^2^, MD Saskia A.G. Stitzinger^2^, MD PhD Maarten-Paul van de Kerkhove^2, 3^, PhD Rianne M. Rozendaal^1^, PhD Marienke van Middelkoop^1^, MD PhD Patrick J.E. Bindels^1^, PhD Sita M.A. Bierma-Zeinstra^1,4^, PhD Dieuwke Schiphof^1^

*^1^Department of General Practice, Erasmus MC University Medical Center, Rotterdam, The Netherlands*

*^2^General Practice Pallion, Hulst, The Netherlands*

*^3^Orthopaedics ZorgSaam Zeeuws-Vlaanderen, Terneuzen, The Netherlands*

*^4^Department of Orthopaedics, Erasmus MC, University Medical Center, Rotterdam, The Netherlands*

**SUPPLEMENTARY TABLE 1:** **interview guides**

**Interview guide: patients**

| Topics | Interview questions |
| --- | --- |
| Experience with the patient-healthcare provider relationship | How did you experience the patient-healthcare provider relationship?  Are you satisfied with the information provided by the healthcare provider(s) regarding your health problem during the appointment? |
| Experienced accessibility of intermediate care; financially, in timing and geographically* | How do you experience the accessibility of intermediate care?   - Experience with waiting times - Experience with travel times - Experience with financial access |
| Experienced difference between usual and intermediate care | What is the difference between primary and intermediate care in your view? |
| Expectation of intermediate care | What were your expectations of intermediate care and does intermediate care comply with your expectations? |
| Other barriers | Have you experienced obstacles in receiving intermediate care? |
| Other facilitators | What are the advantages of intermediate care in your view? |
| Recommendations for improvement of intermediate care | Are there components of intermediate care that you would want to change to make the care better? |

* Accessibility of healthcare can be divided into three aspects:

1. Financially: refers to the extent in which patients are able to pay for healthcare.

2. Timing: refers to the extent in which patients can receive healthcare within a reasonable time (e.g. waiting times).

3. Geographically: refers to the extent in which healthcare facilities are available across the country so that everyone has access the healthcare facility within a reasonable period of time (e.g. travel time and travel distance).

**Interview guide: healthcare providers and healthcare manager**

| Topics | Interview questions |
| --- | --- |
| Start of intermediate care project | How did the intermediate care project start?  Who initiated the intermediate care project?  What were the motivations to start the intermediate care project?  Where there any facilitators/barriers during the start of the intermediate care project? |
| Content of intermediate care | What is the target group of your intermediate care?  What is the protocol for referral of patients to intermediate care?  What is the method of communication between healthcare providers in intermediate care (communication between GPs and orthopaedists)?  What is the duration of an intermediate care consultation?  Are there any facilitators and/or barriers regarding the content of the project? |
| Experienced accessibility of intermediate care; financially and in timing | What is the usual waiting time for patients for a consultation in intermediate care?  Are there any out-of-pocket costs for patients with KHOA who receive a consultation in intermediate care? |
| Experienced difference between usual care and intermediate care | What is the difference in content between intermediate care and usual care (primary and/or secondary care) for patients with KHOA? |
| Financial structure of intermediate care project | How is intermediate care organized financially (for the general practice and patients)?  Are there any facilitators and/or barriers regarding the financial structure of the project? |
| Barriers for intermediate care | Have you experienced obstacles in intermediate care?  Are there components of intermediate care that you would want to change to make the care better? |
| Facilitators for intermediate care | What are the advantages of intermediate care in your view? |
| Recommendations | Do you have recommendations for other intermediate care projects to start up and manage the project successfully? |

**SUPPLEMENTARY TABLE 2: Characteristics of participants participating the semi-structured interviews**

| Role | Female  % (n) | Method of interview |
| --- | --- | --- |
| Orthopaedist (n=2) | 50% (1) | All face-to-face |
| GP (n=4) | 25% (1) | Three face-to-face and one on telephone |
| Patients (n=4) | 25% (1) | All on telephone |
| Healthcare manager (n=1) | 100% (1) | Face-to-face |

**Note.** Age of participants was not reported to preserve confidentiality, as the number of participants within one role is small and increases the likelihood that their confidentiality cannot be guaranteed.

**SUPPLEMENTARY TABLE 3: Content of the intermediate care projects for which semi-structured interviews were carried out**

|  | **Project 1** | **Project 2** |
| --- | --- | --- |
| Target group | Patients with musculoskeletal complaints who would normally be referred to secondary care. | Patients of 50 and older with suspected or confirmed knee and/or hip osteoarthritis; patients with insufficient arguments for surgery; the patients does not sufficiently respond to non-surgical treatment in primary care. |
| Frequency | One afternoon per two weeks. | One evening per two weeks. |
| Length of consultation | 30 minutes | 5-20 minutes |
| Set-up | One-time consultation, normally provided by an orthopaedist alone, sometimes together with a GP. | One-time joint consultation by a GP with special interest and an orthopaedist. |
| Scale of project | One general practice with one orthopaedist. | Two general practices with one orthopaedist for each practice. |
| Method of referral to intermediate care | Referral by a GP. | Referral by a GP, always with an X-ray request. |
| Reasons to start intermediate care project | - Higher quality of care - Lower healthcare costs - More multi-disciplinary collaboration - Intrinsic motivation/ personal interest | - Higher quality of care - Lower healthcare costs - Learning from other disciplines - Providing healthcare in a familiar environment for the patient - Prevent patients from seeking care abroad (Belgium) - Better access to healthcare for the patient - Reducing overuse of hospital care due to unnecessary referrals - More multi-disciplinary collaboration |

**SUPPLEMENTARY FIGURE 1: patients’ satisfaction with intermediate care**

**Practice A**

Table 1. Results of n= 39 patient satisfaction questionnaires on a 4-point Likert scale

**Practice B**

Table 2. Results of n=61 patient satisfaction questionnaires on a scale from 1-10

**Abbreviation:** SD= standard deviation

**SUPPLEMENTARY TABLE 4: Referrals and healthcare consumption in the pre-and post-implementation period stratified by patients who received regular GP care or intermediate care**

|  | **Pre-implementation period** | **Post-implementation period** | |
| --- | --- | --- | --- |
|  | Regular GP care (n=96) | Regular GP care (n=153) | Intermediate care (n=55) |
| Referrals to physiotherapy, n (%) | 5 (5.21) | 21 (50.0) | 21 (50.0) |
| Referrals to orthopaedics, n (%) | 29 (30.2) | 66 (88.0) | 9 (12.0) |
| Number of consultations, mean (SD) | 2.40 (1.59) | 2.55 (1.99) | 2.52 (1.71) |
